# Case Report: Early recognition of neonatal alpha-1 antitrypsin deficiency: a case of subtle presentation and prompt diagnosis

**DOI:** 10.3389/fped.2026.1859034

**Published:** 2026-06-24

**Authors:** Vinson James, Sydney Darling, Catherine Chin, Pomalpreet Bajwa, Adarsh Pillay, Jaime Chu, Yoseph Gurevich, Vindhya Kamath

**Affiliations:** 1 Good Samaritan University Hospital, 1000 Montauk Highway, West Islip, NY, United States; 2 New York Institute of Technology College of Osteopathic Medicine, Old Westbury, NY, United States; 3 Mount Sinai Kravis Children’s Hospital, Mount Sinai’s Recanati/Miller Transplantation Institute, and Icahn School of Medicine at Mount Sinai 10th 5, New York, NY, United States

**Keywords:** alpha-1 antitrypsin deficiency, case report, conjugated hyperbilirubinemia, neonatal cholestasis, neonatal hypothermia

## Abstract

Herein, we present the case of a 4-day-old male infant, born small for gestational age (38 weeks, 2,150 g) to a group B streptococcus-positive mother, admitted with hypothermia, hypoglycemia, and weak cry. Initial evaluation was directed toward neonatal sepsis; however, persistent conjugated hyperbilirubinemia (direct bilirubin peak 2.6 mg/dL; reference <1.0 mg/dL), markedly elevated gamma glutamyl transferase (632 U/L; reference: <70 U/L), and a serum alpha-1 antitrypsin (A1AT) level <20 mg/dL (reference: 100–200 mg/dL) after negative infectious workup prompted metabolic evaluation. The diagnosis of alpha-1 antitrypsin deficiency (PiZZ genotype) was confirmed by the 8th day of life, which is among the earliest reported postnatal diagnoses. An abdominal ultrasound revealed no biliary ductal dilatation but a mildly contracted gallbladder. The condition of the infant improved with supportive management and the patient was discharged with close follow-up with pediatric gastroenterology. This case highlights the importance of considering metabolic and genetic disorders in neonates with persistent conjugated hyperbilirubinemia, particularly after exclusion of infectious causes, and emphasizes that alpha-1 antitrypsin deficiency is not included on routine newborn screening panels.

## Introduction

Neonatal hypothermia and hyperbilirubinemia are common clinical presentations in the first week of life. Although unconjugated hyperbilirubinemia is typically benign and physiologic, conjugated hyperbilirubinemia in the neonatal period warrants urgent evaluation for infectious, metabolic, or structural etiologies. Alpha-1 antitrypsin deficiency (A1ATD) is a rare but important cause of neonatal cholestasis, accounting for up to 10% of neonatal hepatitis cases. The median age of diagnosis for A1ATD cholestasis is 8–12 weeks; diagnosis within the first week of life is uncommon. We describe one of the earliest confirmed cases of neonatal A1ATD diagnosed by the 8th day of life. A review of published series and case reports (1–4) identifies no prior postnatal confirmation earlier than day 7–10; the median reported age at diagnosis ranges from 8 to 12 weeks. This case further highlights the diagnostic limitations of current allele coverage and the absence of A1ATD from standard newborn screening panels.

## Case presentation

A 4-day-old male infant, born at 38 weeks of gestation via normal vaginal delivery, presented with hypothermia. Birth history was significant for intrauterine growth restriction (IUGR and low birth weight (2,150 g, <3rd percentile). The infant’s mother tested positive for group B streptococcus and received appropriate intrapartum IV Penicillin. NICU stay was ruled out, and routine prophylaxis (vitamin K, Erythromycin) was provided; hepatitis B vaccination was declined.

At home, the mother noted low rectal temperatures (94°F–95°F) and weak cry, prompting presentation to the emergency department. An initial evaluation revealed hypothermia (94.4°F), lethargy, and poor feeding. Sepsis evaluation was initiated. A physical examination revealed a term-appearing, small-for-gestational-age male without dysmorphic facial features. The abdomen was soft and non-distended; hepatomegaly was absent on examination. There was no splenomegaly. Jaundice was noted on physical examination. Laboratory workup showed hypoglycemia (glucose: 49 mg/dL; reference: 50–99 mg/dL), elevated liver enzymes [aspartate aminotransferase (AST): 83 U/L, reference: <50 U/L; alkaline phosphatase: 398 U/L, reference: <300 U/L; Alanine aminotransferase (ALT): 34 U/L, reference: <45 U/L; gamma glutamyl transferase (GGT): 632 U/L, reference: <70 U/L], hypoalbuminemia (2.4 g/dL; reference: 3.0–5.0 g/dL), hypoproteinemia (4.7 g/dL; reference: 5.0–7.5 g/dL), total bilirubin: 23.7 mg/dL, and direct bilirubin: 2.4 mg/dL (reference: <1.0 mg/dL). Coagulation studies were obtained because of hypoalbuminemia and cholestasis: the prothrombin time was 14.2 s (INR: 1.3), and the partial thromboplastin time was 36 s. These values did not indicate acute liver failure or vitamin K deficiency bleeding. Cerebrospinal fluid (CSF) was bloody with elevated protein and low glucose levels (49 mg/dL). WBC count was 8,490 /µL. A chest radiograph showed non-specific interstitial prominence, following which broad-spectrum antimicrobials (ampicillin, ceftazidime, acyclovir) were initiated. The initial presentation (hypothermia, hypoglycemia, high unconjugated bilirubin) was consistent with neonatal sepsis and physiological jaundice of the newborn in the setting of IUGR. Conjugated hyperbilirubinemia (peak direct 2.6 mg/dL) and elevated GGT (632 U/L) developed over days 3–5, which pointed to the cholestatic process. A traumatic tap was suspected, and meningitis was excluded upon a negative CSF culture test result and normal CSF gram stain. During admission, the total bilirubin level peaked to 23.7 mg/dL, which decreased after the initiation of triple phototherapy. The culture (blood, urine, CSF) and herpes simplex virus polymerase chain reaction (PCR) test results were negative, leading to discontinuation of antimicrobials. A follow-up of newborn screening showed normal results for all 57 conditions tested in New York State; notably, alpha-1 antitrypsin deficiency is not included on this panel. The direct bilirubin level remained persistently elevated (1.6–2.6 mg/dL) with increased levels of gamma-glutamyl transferase (GGT: 576–632 U/L). Serum alpha-1 antitrypsin (A1AT) level was obtained on the 6th day of life after an infectious workup returned a negative test result and direct hyperbilirubinemia persisted despite phototherapy. The markedly elevated GGT (>500 U/L) with disproportionately normal ALT is a recognized pattern in A1AT deficiency, and in the case of our patient, it prompted targeted A1AT testing ahead of a broader metabolic workup. A1ATD was prioritized as the leading diagnosis given its prevalence as the most common inherited cause of neonatal cholestasis, the persisting direct hyperbilirubinemia without infectious explanation, and the characteristic GGT/ALT dissociation. Liver biopsy was discussed but deferred: the infant showed clinical improvement, phenotyping had established a specific genetic diagnosis, and the risks associated with biopsy in a 6-day-old with borderline coagulation parameters (INR 1.3) were supposed to outweigh the diagnostic benefit at that time. If cholestasis had progressed or the diagnosis remained uncertain, biopsy and intraoperative cholangiography would have been the next step. The serum A1AT level was <20 mg/dL (reference 100–200 mg/dL), confirming severe deficiency. An abdominal ultrasound revealed a mildly contracted gallbladder. Pigmented stools were visualized, but there were episodes of blood-tinged stools attributed to possible protein allergy caused by the consumption of cow's milk, which resolved after maternal dairy elimination and the initiation of a dairy protein hydrolysate formula (Pepticate®). Alpha-1-antitrypsin phenotyping by isoelectric focusing confirmed the PI*ZZ phenotype, which was consistent with homozygous Z-allele deficiency. (Confirmatory PCR-based genotyping was not performed at our institution; The PI*ZZ status was therefore established by phenotypic analysis.)

Additional diagnostic evaluation to exclude other causes of neonatal cholestasis:

- Cytomegalovirus (CMV) testing: the result of a urine CMV PCR was negative; maternal CMV serology (IgG positive, IgM negative) indicated past infection without active congenital infection.
- Cystic fibrosis was excluded because of a normal immunoreactive trypsinogen level on newborn screening and a negative sweat chloride test result (12 mEq/L; normal <30).
- Alagille syndrome was considered less likely due to the absence of characteristic facial features, posterior embryotoxon on ophthalmologic examination, butterfly vertebrae on a skeletal survey, and a normal echocardiogram.
- Total serum bile acid levels were elevated at 156 µmol/L (normal: <10), consistent with cholestasis but non-diagnostic for a specific etiology.
- Family history was non-contributory. The parents of the infant were non-consanguineous and of mixed European descent. There was no family history of liver disease, chronic obstructive pulmonary disease (COPD), panniculitis, or early-onset emphysema. The mother had no history of miscarriages or unexplained neonatal deaths. Parental carrier testing was initiated as part of the diagnostic workup. The mother underwent A1AT phenotyping and was found to carry the PI*MZ phenotype, confirming her status as an obligate carrier of one Z allele. The father declined testing during the acute hospitalization period; outpatient genetic counseling was recommended for paternal testing and extended family screening.

**Figure 1:**
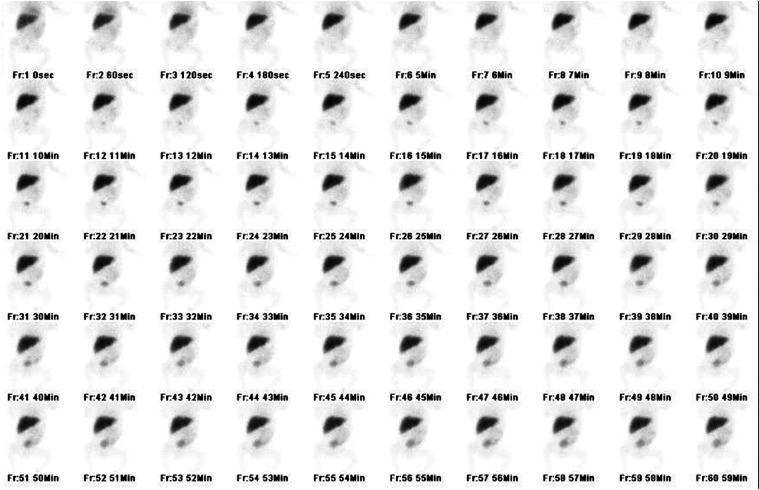
HIDA scan 1.

**Figure 2:**
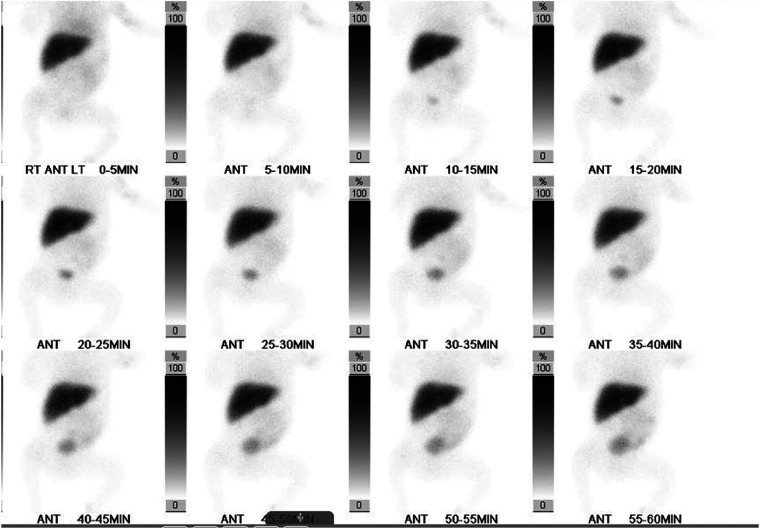
HIDA scan 2.

Blood, CSF, and urine cultures showed no growth. The infant was discharged on the 9th day of life with stable vital signs, adequate feeding, and persistent direct hyperbilirubinemia. Outpatient follow-up with pediatric hepatology was arranged for ongoing management of alpha-1 antitrypsin deficiency. Ursodeoxycholic acid 15 mg/kg/day was initiated. Fat-soluble vitamin levels (A, D, E, and K) were not obtained during the acute admission period because of the short duration of hospitalization; outpatient monitoring was arranged. Cholestasis persisted and the direct bilirubin level was 2.1 mg/dL at 4 weeks of life. A hepatobiliary iminodiacetic acid (HIDA) scan was deferred during acute admission on clinical grounds: pigmented stools were documented throughout the period of hospitalization, an abdominal ultrasound demonstrated a visible gallbladder without biliary ductal dilatation, and the PI*ZZ phenotype had been confirmed by day 8. Importantly, even a HIDA scan demonstrating biliary excretion would not have excluded biliary atresia (BA) with a level certainty to alter management—if cholestasis had progressed, liver biopsy and intraoperative cholangiography would have been pursued regardless. The HIDA scan was therefore deferred to the outpatient setting, where clinical trajectory could be reassessed before committing to invasive evaluation. Following hospital discharge, the (HIDA) scan revealed the excretion of a radiotracer into the bowel, making biliary atresia less likely (Figures 1, 2). We acknowledge that intraoperative cholangiography remains the definitive gold standard for excluding biliary atresia, which, however, was not pursued in this patient given the clinical improvement and alternative diagnosis (Table 1).

**Table 1:** Diagnostic timeline of clinical events.

Day of life	Symptoms/signs	Key laboratory values	Interventions
1 (birth)	Born at 38 weeks, IUGR, weight 2,150 g	Routine newborn screen (normal)	Vitamin K and erythromycin
4	Hypothermia (94.4°F), weak cry, poor feeding, and lethargy	Glucose 49 mg/dL, TB 23.7 mg/dL, DB 2.4 mg/dL, AST 83 U/L, ALT 34 U/L, GGT 632 U/L, and A1AT <20 mg/dL	Sepsis workup, ampicillin + ceftazidime + acyclovir, and triple phototherapy
5–6	Persistent jaundice and direct hyperbilirubinemia	PT 14.2 s (INR 1.3) and PTT 36 s; all cultures negative; urine CMV PCR negative	Antimicrobials discontinued; A1AT testing sent
7	Stable, and feeding improved	Phenotype confirmed PiZZ	Ursodeoxycholic acid 15 mg/kg/day started
8–9	Discharged with persistent DB 2.1 mg/dL	Sweat chloride 12 mEq/L (normal); bile acids 156 µmol/L	Outpatient hepatology follow-up arranged
28 (4 weeks)	Follow-up	DB 2.1 mg/dL, and continued elevation of liver enzymes	Ongoing ursodeoxycholic acid; an HIDA scan performed (excretion into bowel, BA less likely)

## Discussion

A1AT deficiency represents an important differential diagnosis when evaluating pediatric liver disease because of its heterogeneous clinical course and overlap with other cholestatic conditions. In neonates, A1AT deficiency can present with prolonged cholestatic jaundice within the first few weeks of life. Laboratory evaluation typically demonstrates conjugated hyperbilirubinemia, elevated transaminases, and variable coagulopathy. Clinically, early presentation can mimic other causes of neonatal cholestasis, including biliary atresia. The case of the patient in this study is notable for diagnosis by the 8th day of life, which is significantly earlier than the median age of 8–12 weeks reported in large series. The disease course varies widely—some infants recover from cholestasis within the first year, while others develop chronic liver damage leading to fibrosis, cirrhosis, or portal hypertension, sometimes requiring liver transplantation in infancy. This unpredictability underscores the importance of close longitudinal follow-up.

### Why this case is unique

Unlike typical A1ATD presentations with isolated cholestasis, our patient initially presented with hypothermia, hypoglycemia, weak cry, and severe unconjugated hyperbilirubinemia—features that strongly suggested sepsis. The early metabolic derangements (hypoglycemia, hypoalbuminemia) were likely caused by the combined effects of IUGR, poor feeding, and hypothermia, while cholestasis (direct bilirubin: 2.6 mg/dL, GGT: 632 U/L) was the manifestation of A1ATD. Normal ALT (34 U/L) is considered unusual for A1ATD hepatitis, but the literature shows that ALT can be normal in early neonatal A1ATD cholestasis (5).

### Diagnostic pitfalls

First, A1ATD is not included on the New York State newborn screening panel nor on the Recommended Uniform Screening Panel in the United States. Second, the absence of family history does not exclude A1ATD because of mechanisms such as silent heterozygosity, *de novo* mutations, and uniparental disomy. Third, rare variants (non-S/non-Z) account for up to 10% of pathogenic SERPINA1 alleles and are missed by standard genotyping.

### Differential diagnosis of neonatal cholestasis in our patient

The differential diagnosis for neonatal cholestasis is broad and includes biliary atresia, infectious etiologies, metabolic disorders, and endocrine causes. Biliary atresia, the most urgent diagnosis to exclude, is suggested by progressive jaundice, pale stools, hepatomegaly, and characteristic ultrasonographic findings. In the case of the patient in this study, an abdominal ultrasonography demonstrated a normally visualized gallbladder with no biliary ductal obstruction, possibly ruling out biliary atresia. Infectious causes such as TORCH infections, urinary tract infection, and neonatal sepsis were considered; however, the infant had normal inflammatory markers and negative blood and urine cultures, effectively excluding infectious cholestasis. Metabolic etiologies such as galactosemia, tyrosinemia, and bile acid synthesis defects were evaluated, and the newborn screening results, amino acid profiles, and the absence of hypoglycemia or coagulopathy probably ruled out the presence of these etiologies. Endocrine causes, particularly congenital hypothyroidism and adrenal insufficiency, were ruled out after conducting normal thyroid function tests, and there was no clinical or biochemical evidence of cortisol deficiency. Importantly, in view of persistent direct hyperbilirubinemia, alpha-1 antitrypsin (A1AT) tests were performed, which showed low serum levels with a PiZZ phenotype, confirming A1AT deficiency as the underlying etiology in this case.

Approximately 90% of normal individuals exhibit the MM phenotype, associated with normal quantitative levels of A1AT. Numerous phenotypic variants have been described, including deficiency alleles such as S, Z, F, and others. Relative to the normal M allele, which produces 100% of functional A1AT, the S allele yields approximately 60%, and the Z allele approximately 20% of normal protein levels. Thus, individuals with the MS genotype typically produce approximately 80% of normal A1AT (≈50% from the M allele and ≈30% from the S allele), whereas those with the ZZ genotype produce only 20% of normal levels (≈10% from each Z allele) (1, 6–8). The F allele produces near-normal quantities of A1AT but demonstrates reduced elastase inhibitory activity, rendering it functionally mildly deficient. A phenotypic analysis identified numerous other variants—such as CM, DP, EM, GM, IS, LM, M1M2, M3M3, MP, MT, XX, MY, and M1N. Among these, I, P, T, and null alleles are considered deleterious, whereas C, D, E, G, L, M1, M2, M3, X, and Y alleles are generally regarded as normal variants. The MZ-Pratt phenotype is a benign variant and should not be mistaken for the deficient MZ phenotype (6, 7).

Importantly, A1AT deficiency also has well-recognized pulmonary manifestations, particularly in adults, but early-life exposure to risk factors such as second-hand smoke has been linked to worsened outcomes, highlighting the importance of preventive counseling even in pediatric patients. At present, there are no interventional clinical trials specifically targeting pediatric A1AT deficiency. Although affected children may be enrolled in observational registries, therapeutic studies remain limited to adults, with the expectation that pediatric participation will expand as future trials are developed. In our patient, early identification allowed timely referral and nutritional management, which are critical in preventing progression to hepatic injury. This case illustrates the need for broader genotyping and awareness of atypical neonatal presentations of A1ATD to prevent delayed diagnosis.

### Absence of family history

The absence of a positive family history does not exclude A1ATD. Published cases document PI*ZZ diagnoses in individuals with no prior family history of liver disease, COPD, or emphysema—explained by mechanisms such as silent heterozygosity in unaffected parents, *de novo* mutations, and rare events such as maternal uniparental disomy of chromosome 14 (UPD14mat), which can unmask a maternally inherited Z allele even when paternal genotyping is M/M (1, 6, 7). As in our case, the parents' apparently unremarkable pedigree and the mother's carrier status (PI*MZ) were not apparent until directed genetic testing was performed; the father remained untested at discharge, underscoring the need for systematic family screening following every pediatric A1ATD diagnosis.

### Diagnostic and genetic limitations of newborn screening

In New York State, newborn screening currently includes 57 conditions, encompassing metabolic, endocrine, and hematologic disorders. However, A1ATD is not part of the state's mandated newborn screening panel. Diagnosis therefore depends on clinical recognition rather than population screening, resulting in many missed or delayed identifications of affected infants (8). Because A1ATD is not routinely screened, diagnosis typically occurs after clinical symptoms develop, often years later. Evidence shows that the PI*ZZ genotype, the most severe form of deficiency, is frequently underrecognized or misdiagnosed by clinicians. Newborns with a family history of early-onset emphysema (before age 45), emphysema in non-smokers, basilar hyperlucency, unexplained liver disease, necrotizing panniculitis, antiproteinase 3-positive vasculitis, or familial clustering of liver or pulmonary disease should be considered for screening for alpha-1 antitrypsin deficiency when clinically indicated (9). The diagnostic process usually begins with the measurement of serum A1AT levels, followed by phenotyping or genotyping in specialized laboratories when values are low or when clinical suspicion remains high. In inconclusive cases, gene sequencing may be necessary to confirm the diagnosis, and patients should be referred to centers with expertise in A1ATD management (10).

Despite the identification of *α*1-antitrypsin deficiency (A1ATD) through newborn screening, there is currently no evidence that early detection alters neonatal liver outcomes, as the liver disease associated with A1ATD often presents later in childhood and lacks specific treatments beyond supportive care and liver transplantation. Early identification of alpha-1 antitrypsin deficiency not only enables preventive strategies to reduce future risk of chronic lung disease, particularly COPD, through behavioral interventions such as smoking avoidance, but also provides a clear etiology for neonatal cholestasis—thereby reducing the need for extensive or invasive evaluation for biliary atresia. However, this represents a substantial shift in public health screening philosophy, as it targets disease prevention decades after the neonatal period, and its effectiveness remains uncertain (11).

### Public health implications of current allele coverage and missed variants

Standard A1ATD genotyping panels target only the common S and Z variants of the SERPINA1 gene; more than 200 additional pathogenic variants remain undetected by conventional assays, contributing to diagnostic delays that often span several years (12, 13). Clinical uptake of expanded testing remains low, particularly among non-White populations and current smokers (2, 14). A1ATD is not part of routine universal newborn screening in any US state; screening for A1ATD remains limited to targeted pilot studies and research initiatives rather than standard state panels. As a result, neonatal A1ATD cases may be missed by routine newborn screening and are typically identified only after clinical presentation or directed testing (11, 15).

### Typical course in neonatal A1ATD (PiZZ phenotype)

- Cholestasis phase: Many affected infants show direct hyperbilirubinemia and elevated ALT/AST/GGT during the first few weeks to months of life.
- HIDA scans often show biliary excretion into the bowel (ruling out biliary atresia) but with persistent hepatic retention, reflecting hepatocellular dysfunction and cholestasis rather than obstruction (2).
- Evolution after diagnosis: Over weeks to months, bilirubin levels gradually decline, although mild transaminitis and GGT elevation can persist for several months. Approximately 80%–85% of infants show spontaneous improvement of cholestasis by 1 year of age, while 10%–15% may progress to fibrosis, cirrhosis, or portal hypertension (16).
- Long-term implication: Even after normalization of bilirubin, mild biochemical abnormalities may persist, and therefore, continued pediatric hepatology follow-up is the standard treatment (17).

### Evaluation for biliary atresia despite identification of alpha-1 antitrypsin deficiency

Although A1ATD is a well-recognized cause of neonatal cholestasis, BA remains the most critical differential diagnosis because of the narrow therapeutic window for successful portoenterostomy. The coexistence of A1ATD and BA, although rare, has been reported in the literature, underscoring the importance of not excluding BA solely based on an established diagnosis of A1ATD (18–20). Therefore, standard evaluation with hepatobiliary scintigraphy (HIDA scan) and, when appropriate, intraoperative cholangiography, remains essential in all neonates with conjugated hyperbilirubinemia (21). In the case of the patient in this study, the HIDA scan confirmed the excretion of a tracer into the bowel, effectively ruling out BA and guiding the clinical team toward a metabolic etiology. This diagnostic vigilance ensures that treatable obstructive causes are not overlooked, even when a genetic etiology is identified early.

### The role of the Z allele as a modifier of liver disease severity

The clinical expression of A1ATD-associated liver disease is highly variable even among PI*ZZ homozygotes, reflecting the influence of modifier genes, epigenetic factors, and environmental exposures (22–26). Approximately 80%–85% of affected infants show spontaneous resolution of cholestasis within the first year; 10%–15% progress to fibrosis or cirrhosis (24). Heterozygous PI*MZ carriers—such as the mother in this case—may also carry increased susceptibility to chronic liver injury, reinforcing the importance of long-term follow-up across the family (27).

### Practical algorithm for clinicians

For any neonate with direct bilirubin >1.0 mg/dL persisting beyond 14 days of life, we recommend the following:

1. Exclude biliary atresia (ultrasound, consider HIDA, refer for cholangiography if there is no improvement).
2. Exclude infection (blood, urine, CSF cultures; CMV PCR; TORCH panel).
3. Obtain serum A1AT levels (low <50 mg/dL suggests deficiency; confirm with phenotype/genotype).
4. Consider CF, Alagille, and bile acid defects based on clinical features.

## Conclusion

Although most neonatal cases of alpha-1-antitrypsin deficiency are diagnosed after the onset of cholestasis or bleeding during the second to fourth postnatal week, our case represents one of the earliest reported postnatal diagnoses, established within the first 8 days of life (3, 28, 33). Given that A1ATD is not part of standard newborn screening panels in the United States or Europe, early identification in this instance underscores the importance of considering A1ATD in the differential diagnosis of unexplained neonatal liver dysfunction (1, 4). In neonates, hypothermia and cholestasis are non-specific clinical findings that frequently point toward systemic infection, particularly sepsis or TORCH infections. Therefore, infectious etiologies are typically the first consideration and must be excluded promptly because of their potential for rapid deterioration and the need for urgent antimicrobial therapy (29, 32).

However, when infectious causes are ruled out, persistent cholestasis with hypothermia should prompt evaluation for metabolic and genetic disorders, as these conditions often present with overlapping features in early life. A1ATD, one of the most common inherited causes of neonatal cholestasis, may initially mimic sepsis or hepatitis of infectious origin. Unlike infection, A1ATD results from impaired secretion of misfolded alpha-1 antitrypsin in hepatocytes, leading to hepatocellular injury and cholestasis (30, 31).

Recognizing this possibility early allows for appropriate diagnostic testing (e.g., serum A1AT levels and phenotype/genotype analysis) and family counseling, preventing unnecessary antibiotic exposure and aiding long-term management (5, 23). Thus, maintaining a high index of suspicion for metabolic and genetic disorders like A1ATD is crucial when evaluating neonates with unexplained hypothermia and cholestasis after infection has been excluded. A limitation of this report is the short follow-up period of 4 weeks; extended longitudinal data on bilirubin normalization, growth parameters, liver function, and hepatology outcomes were not available at the time of submission and represent an area of future research (32, 33).

## Data Availability

The original contributions presented in the study are included in the article/Supplementary Material, and further inquiries can be directed to the corresponding author.
